# Multidimensional Study of the Attitude towards Euthanasia of Older Adults with Mixed Anxiety-Depressive Disorder

**DOI:** 10.1192/j.eurpsy.2025.458

**Published:** 2025-08-26

**Authors:** L. Fonseca, G. Rêgo, R. Nunes

**Affiliations:** 1 Bioethics, Faculty of Medicine, University of Porto, Porto; 2 Psychiatry, Senhora da Oliveira Hospital, Guimarães, Portugal

## Abstract

**Introduction:**

Euthanasia is an ancient theme that, especially since individual autonomy became the health paradigm in contemporary societies, has sparked profound reflections and declared dissensions between different socio-ideological quadrants. The experience of the countries where it is decriminalised shows a tendency to broaden the clinical, age and legal assumptions for its access. Older adults with psychiatric disease, a clinically and socially idiosyncratic group, where physiological weaknesses and social losses accumulate, and the chronological proximity to death becomes progressively more self-aware, are a group of particular concern.

**Objectives:**

Our research aimed to reflect on the Constitutional feasibility of Euthanasia in Portugal, make available a validated psychometric instrument to assess attitudes towards euthanasia and do a multidimensional study of the attitudes towards euthanasia of older adult patients with mixed anxiety-depressive disorder.

**Methods:**

The field research study applied a paper questionnaire composed of a sociodemographic section and a battery of scales (to assess depression, anxiety, cognitive performance, suicide risk, therapeutic adhesion, functionality, loneliness, attitude towards euthanasia, decision pattern, personality, empathy and health status) in the Psychogeriatric Unity of Senhora da Oliveira Hospital in Portugal. The sample was collected by convenience. The multidimensional study included 114 patients and 25 controls of the same age. Six months later, a reassessment was conducted. Patients and controls were compared using descriptive statistics and a multiple-regression model.

**Results:**

The Constitution of the Portuguese Republic does not prohibit medically assisted death. The results support the validated scale’s usefulness and validity. Eighty-one point six per cent of patients had four or fewer years of schooling. Contrary to controls, they presented mild depressive and anxiety symptoms, loneliness feelings, worse cognitive performance, a more fragile personality, higher personal distress and a poorer health state. No statistically significant differences were found between controls and patients regarding their attitudes towards euthanasia. Patients more favourable to euthanasia had higher empathic concern, conscientiousness and fantasy and lower personal distress.

**Conclusions:**

When addressing euthanasia in older adult patients with mixed anxiety and depressive disorder, it is crucial to ensure they are fully self-determinate and that all the necessary treatment and support are available. It may not be the case when the educational level is low and mild disease persists, significantly affecting their well-being and cognitive performance.

**Disclosure of Interest:**

None Declared

